# Efficacy and tolerability of Profhilo® Structura intended to restore lateral cheek fat compartment: An observational pilot study

**DOI:** 10.1002/hsr2.1743

**Published:** 2024-01-21

**Authors:** Adele Sparavigna, Franco Grimolizzi, Clara Cigni, Roberto Lualdi, Gilberto Bellia

**Affiliations:** ^1^ DERMING S.r.l. Milano Italy; ^2^ IBSA Farmaceutici Italia Lodi Italy

**Keywords:** face fat compartment, hyaluronic acid, hybrid cooperative complexes, safety

## Abstract

**Background and Aims:**

Hyaluronic acid (HA)‐based injections are used worldwide to improve skin defects associated with aging and ultraviolet light/environmental exposure. HA formulations developed according to molecular weight or with additional components, for example, cross‐linking reagents, are limited by their low biological activity and concentration limit. NAHYCO™ technology has enabled the production of hybrid cooperative complexes (HCCs) of low and high molecular weight HA. Developed for injection into the fat compartments of the face and previously demonstrating potential benefits for adipose tissue restoration, Profhilo Structura® is a new 2 mL HCC formulation comprising low molecular weight HA (45 mg/mL) and high molecular weight HA (45 mg/mL). To evaluate the efficacy and tolerability of Profhilo Structura® to restore adipose tissue compartments in the lateral cheek fat compartment.

**Methods:**

Fifty healthy enrolled subjects received two injections, 1 month apart, and were evaluated 3 months posttreatment. Investigators performed clinical evaluations (Facial Volume Loss Scale [FVLS] and Wrinkle Severity Rating Scale [WSRS]) at different time points. Subjects also completed self‐evaluation assessments following treatment.

**Results:**

A significant improvement in FVLS and WSRS clinical scores after the first treatment was observed; treatment benefit was maintained 3 months after treatment completion and confirmed by subject self‐assessment. Most participants reported an improvement, particularly a marked reduction of wrinkles and increased skin firmness. No serious adverse events were reported, confirming the excellent safety profile of HCC injectable devices.

**Conclusions:**

Overall, the study highlighted the efficacy and tolerability of the studied medical device proving its effect on adipose tissue.

## 1 INTRODUCTION

Skin aging and facial age‐related morphological changes are usually related to the loss of skin elasticity due to intrinsic (e.g., genetic) and extrinsic (e.g., sun exposure, smoke, and lifestyle) factors. Morphological changes occurring during aging lead to a loss in facial volume and the formation of wrinkles.^1^, ^2^, ^3^, ^4^, ^5^ These appearance modifications can cause some people distress and have a marked impact on social behavior and relationship, leading to an overall increase in esthetic procedures.^6^, ^7^, ^8^, ^9^

Increasing evidence suggests that adipose tissue can represent a suitable target in esthetic medicine. Fat compartments in the face can be anatomically categorized as superficial (e.g., nasolabial fat, superficial medial cheek fat, and intraorbital fat) or deep (e.g., buccal fat, deep medial cheek fat, and medial and lateral suborbicularis oculi fat). The fat compartment plays an important role in midfacial aging.^10^, ^11^, ^12^ A decrease in the fat compartment of the face has been well‐documented with age.^13^, ^14^ Moreover, a decrease in the mean size of adipocytes has been found in the deep cheek fat compartment compared to the superficial one in elderly subjects compared with young people.^15^ For these reasons, several treatment options for facial rejuvenation have been considered in the last few years involving fat compartments, including lipotransfer.^16^ However, the use of this technique is still limited by its invasiveness and unpredictable results.

Hyaluronic acid (HA) treatments are well‐known and used for facial rejuvenation and esthetic procedures due to the ability of this polymer to reduce skin aging and wrinkle formation.^17^, ^18^, ^19^ As a result of its specific biochemical and physical characteristics, HA is one of the most widely used components of fillers worldwide,^20^, ^21^ and can be administered in different formulations, depending on HA molecular weight, composition, and concentration. However, several limitations are known for HA fillers, such as the use of additional chemical reagents, its short half‐life, and its concentration limit.

Patented NAHYCO® technology enabled the delivery of a high concentration of HA. Several studies demonstrated that HCC had a longer resistance to degradation.^22^ versus traditional HA treatments and showed efficacy in ameliorating esthetic defects due to wrinkles.^23^ HCC also proved effective for sustaining the vitality of human adipose stem cells and adipocytes and maintaining the fat compartment.^24^ A new formulation of HCC, Profhilo Structura®, has been developed for adipose tissue restoration. Profhilo Structura® contains a higher concentration of HA (45 mg of low molecular weight HA plus 45 mg of high molecular weight HA in a 2 mL injection) and different rheological properties compared with the previously commercialized Profhilo® (32 mg of low molecular weight HA plus 32 mg of high molecular weight HA in a 2 mL injection) product. As a result of these properties, Profhilo Structura® is a product designed for restoring face adipose tissue compartments. This study aimed to further evaluate the efficacy and safety of Profhilo Structura® after injection in the lateral cheek fat compartment of the face via a cannula in healthy participants.

## 2 MATERIALS AND METHODS

### 2.1 Inclusion criteria for the study

The study included 50 Caucasian participants requesting face volume restoration who presented with skin laxity in the lateral cheek fat compartment intended as FVLS and WSRS score ranging from 2 to 4 and agreed to avoid direct exposure to ultraviolet light without appropriate protection for the entire study. Exclusion criteria included pregnancy, lactation, chronic or skin‐related pathologies, drug administration, and heavy smokers. Enrolled subjects were also required to have not performed any other treatment or surgical procedure in the target treatment area within 6 months before study enrollment. The study was approved by an Independent Ethics Committee.

### 2.2 Study design

**Figure 1:**
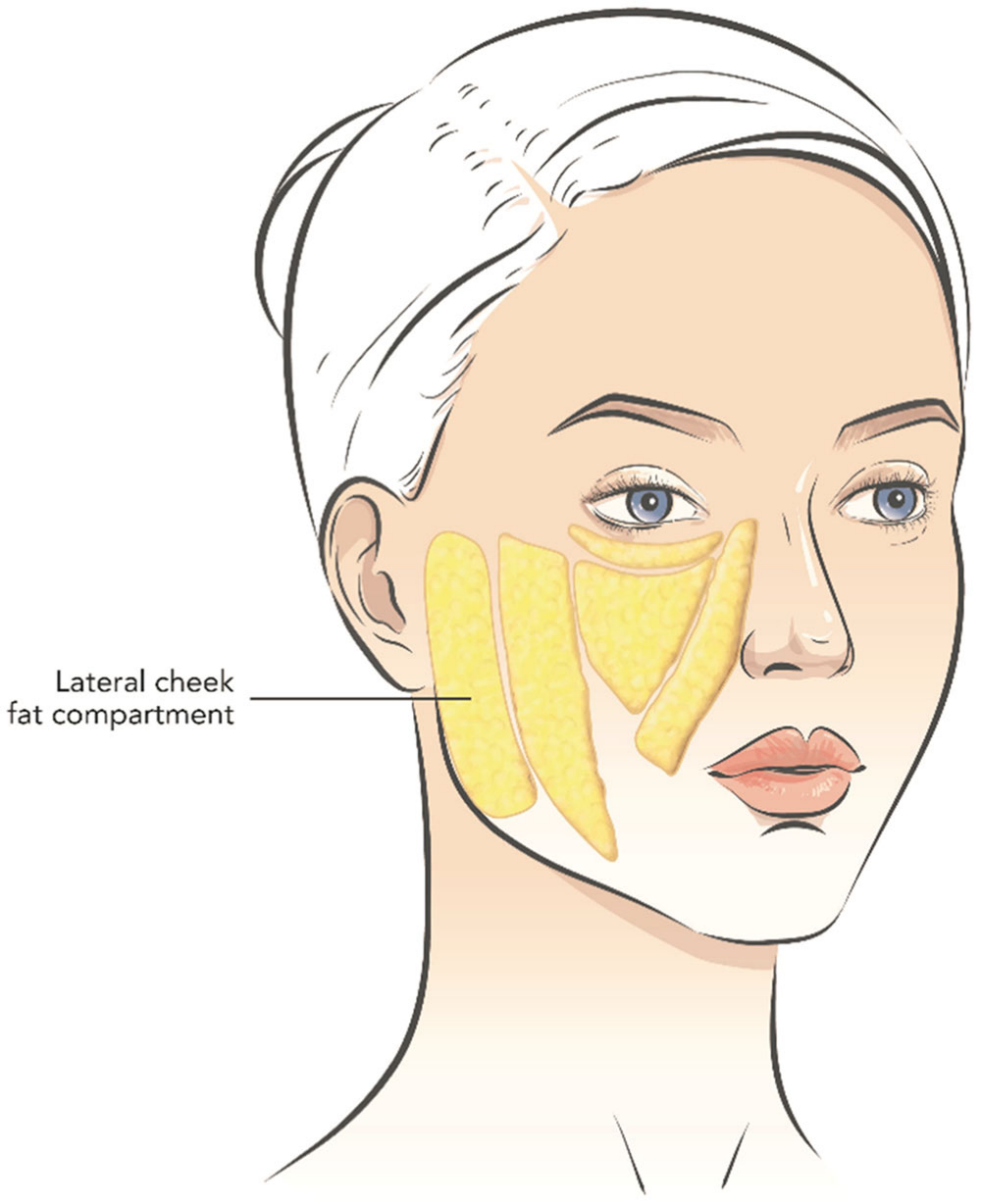
Schematic representation of human adipose tissue compartments in face. Profhilo Structura® was injected using a 25G × 50 mm cannula into the preauricular area (retrograde injection, 1 mL, entry point 2 cm from the tragus), corresponding to the superficial lateral cheek fat compartment.

The single‐center study was conducted under the supervision of a specialized dermatologist. Subjects were treated with Profhilo Structura® (IBSA Farmaceutici Italia Srl) injected into the lateral cheek fat compartment (Figure 1). More specifically, treatments were injected in the superficial fat compartment of the preauricular area, using a 25G × 50 mm cannula (retrograde injection, 1 passage, 1 mL for each treatment) using a single‐entry point 2 cm from the tragus. Participants were treated twice within 30 days and had a follow‐up visit 3 months after the second treatment. The study design included three visits: a visit at baseline (before treatment administration and for the first injection procedure; T0), a subsequent visit for the second injection procedure (1 month after the first injection; T1), and a final follow‐up visit (3 months after the end of the treatment; T2).

### 2.3 Clinical evaluation

The investigators performed clinical evaluations at each time point (T0, T1, and T2). The Facial Volume Loss Scale (FVLS) and Wrinkle Severity Rating Scale (WSRS) were used during the three visits to evaluate potential amelioration after treatment. FVLS evaluates the severity of skin folds or creases and volume loss in different facial areas via a scale representing different severity levels. Similarly, WSRS evaluates the severity of wrinkles using a scale ranging from the absence of wrinkles to very severe wrinkles. Each scale comprises five different grades, ranging from 1 (best appearance) to 5 (worst appearance).

For all the subjects, 3D images of the face were taken using the VECTRA H1 Canfield imaging system. The photos were taken under standardized conditions to allow for image comparison, particularly concerning the distance from the subject and the intensity of the illumination source. During photo execution, the subjects were asked to keep still, with their eyes open, and to relax their facial muscles. Participants completed a self‐assessment questionnaire at the end of the study (T2) to evaluate their overall satisfaction after treatment regarding wrinkle reduction, lifting effect, improvement of skin quality (firmness, smoothness, brightness, and hydration), and reshaping of face silhouette.

### 2.4 Safety assessment

Local reactions (i.e., tardive swelling, pain, erythema, or bruising) and any other local or systemic adverse event or reaction were monitored at each visit and after both procedures to assess product safety. The safety assessment was performed by the investigators, and participants also completed a self‐evaluation assessment at the end of the study.

### 2.5 Sample size calculation

The sample size was calculated based on the incidence of total adverse events (TAEs) over the investigation, considering that a minimum of 35 patients was required for the study enrollment. The probability that one or more TAE did not occur in a sample of 35 patients with an anticipated incidence rate of TAE of 15% was 5% (the power of this investigation was 95% in such situation).

The enrollment of a total of 50 patients would have accounted for the replacement of up to 20% of non‐evaluable patients for any reason.

### 2.6 Statistical analysis

Baseline characteristics were calculated using descriptive statistics (median and range). Statistical analyses were performed in accordance with STROBE guidelines and with standard procedures (descriptive and inferential analysis).

First, a parametric unpaired *t* test (two‐tailed) was used to determine differences between two groups (T1 vs. T0; T2 vs. T0; T2 vs. T1). Then, when statistically significant differences were found, Friedman's test was performed (*k* = 3; *n* = 50) to confirm the obtained results. GraphPad software v10 was used for statistical analysis. The statistical analysis included all the participants' data for each time point, as no drop‐out occurred.

## 3 RESULTS

**Figure 2:**
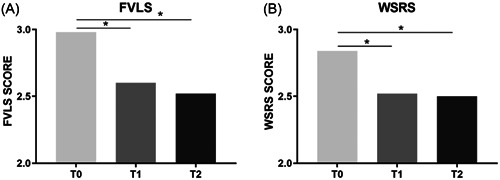
FVLS (A) and WSRS (B) score at baseline visit (T0) and after one or four months after the first treatment (T1 and T2, respectively). *p < 0.05 calculated as nonparametric test using FVLS and WSRS median values (T1 or T2 vs. T0). FVLS, Facial Volume Loss Scale; WSRS, Wrinkle Severity Rating Scale.

This single‐center study enrolled 50 female participants, with no participant drop‐out during the study. The mean age (range) of participants in the study was 54 (40−70) years old. FVLS results showed a significant improvement 30 days after T1 or first treatment (FVLS range at T1 [2.4 – 2.7] vs. T0 [2.8–3.2], *p* < 0.05) with a further and statistically significant amelioration at T2 (FVLS range at T2 [2.3–2.6] vs. T0 [2.8–3.2], *p* < 0.05), 3 months after the end of the treatment (Figure 2A). For further evaluation, an improvement in FVLS severity by at least 1 grade was considered clinically significant for the treated subjects. Interestingly, this 1‐grade improvement was detected in 36% and 64% of the subjects one (T1) and four (T2) months after the beginning of the treatment, respectively.

**Figure 3:**
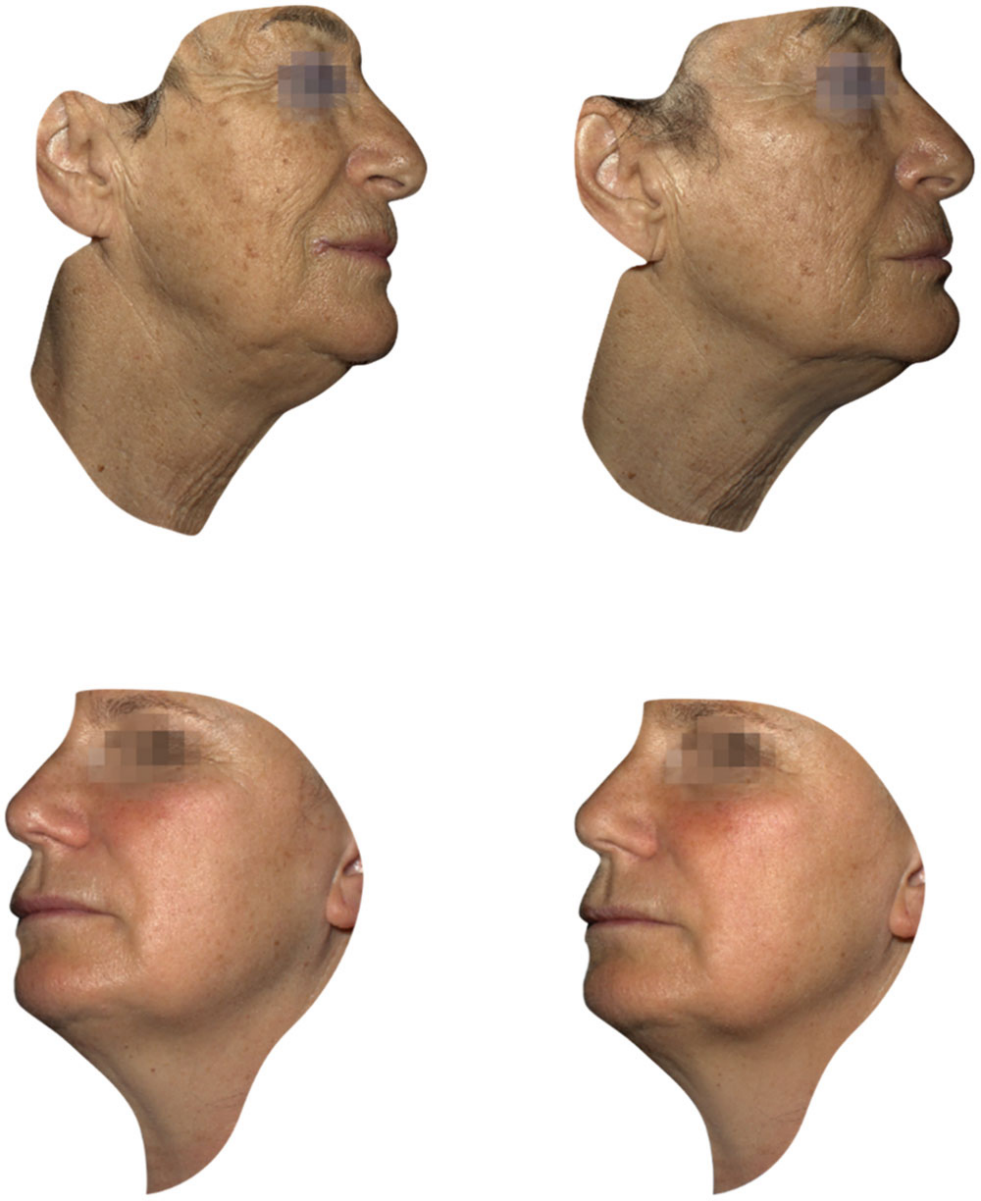
Representative 3D photographic documentation taken at baseline visit or T0 (left side) and 4 months after the first treatment or T2 (right side).

The treatment further demonstrated its efficacy with a significant improvement in WSRS. Similar to FVLS results, subjects showed a statistically significant (WSRS range at T1 [2.3–2.6] vs. T0 [2.7–3.1], *p* < 0.05) improvement 30 days after treatment (T1) with a further WSRS reduction 3 months (T2) after the end of the treatment (WSRS range at T2 [2.2–2.6] vs. T0 [2.7–3.1], *p* < 0.05) (Figure 2B). An improvement by at least 1 grade of the scale was observed in 38% and 51% of the subjects 1 (T1) month and 4 (T2) months after the beginning of treatment, respectively. Clinical evaluations using 3D photographic documentation (example shown in Figure 3) demonstrated a clear improvement of the skin lifting effect which was still evident 3 months after the end of the treatment (T2), particularly regarding facial volume loss in the lateral cheek fat compartment.

Clinical evaluations from the investigators were also confirmed by subject self‐assessments conducted at the end of the study (Table 1). All subjects reported a positive outcome after treatment and light to very marked amelioration in appearance for all the parameters. Notably, all subjects reported a clear improvement in skin firmness, of whom 54% of the participants reported a marked or very marked improvement after treatment with the studied medical device. Furthermore, 84% and 74% of the subjects reported a strong amelioration (from medium to very marked) for the appearance of deep and superficial wrinkles, respectively (Table 1).

**Table 1:** Efficacy evaluation by participants performed at the end of the study (T2).

Parameter	% Of subjects reporting amelioration				
	Type of amelioration				
	Absent	Light	Medium	Marked	Very Marked
Reduction of deep wrinkles	0	16	46	4	34
Reduction of superficial wrinkles	0	26	44	6	24
Lifting effect	0	40	52	8	0
Improvement of skin firmness	0	0	46	50	4
Improvement of skin smoothness	0	40	56	2	2
Improvement of skin brightness	0	40	54	6	0
Improvement of skin hydration	0	44	54	2	0
Reshaping of face silhouette	0	44	50	6	0

Regarding the safety and tolerability of the medical device, the investigators only reported the appearance of light bruises at the injection sites in 4 of 50 subjects (Table 2). These reactions, which completely disappeared within 5−10 days, represented expected events due to the injection procedure alone and were deemed unrelated to the product. No unexpected serious adverse events, adverse device effects, or serious adverse device effects were reported during the study. For these reasons, the investigators judged the product tolerance to be good or excellent in 89% and 11% of subjects, respectively. This outcome was also confirmed by the subject self‐assessment performed at the end of the study, where 68% and 24% of the subjects reported excellent and good tolerance to the treatment, respectively (Table 3).

**Table 2:** Safety evaluated by the investigator during treatment.

Type of event	Number of subjects	%
None	46	92
Expected		
Light bruises	4	8
Small bump	0	0
Unexpected		
SAE	0	0
ADE	0	0
SADE	0	0

Abbreviations: ADE, adverse device effect; SADE, serious adverse device effect; SAE, serious adverse event.

**Table 3:** Tolerance evaluated by subjects 3 months after the end of the treatment (T2).

Tolerance	% Of subjects
Excellent	68
Good	24
Medium	8
Poor	0
Bad	0

## 4 DISCUSSION

There is an increasing demand for esthetic treatment to counteract morphological changes that occur during the aging process. For this reason, several HA‐based injectables have been developed during the last decade, and strong efforts are currently ongoing to increase their efficacy and tolerability by preparation of new formulations. The patented NAHYCO® technology enables the delivery of HCC comprising low and high molecular weight HA characterized by increased stability and resistance to hyaluronidase degradation,^22^ and its ability to sustain skin cell vitality and proliferation.^22^, ^24^ Moreover, HCC (commercial name, Profhilo®) demonstrated efficacy in clinical studies for improving facial appearance^25^ and counteracting skin laxity^26^ with a high safety profile.^27^

This monocentric study aimed to evaluate the efficacy and tolerability of Profhilo Structura® (a new formulation containing a higher concentration of HA; 45 mg/mL) injected into the lateral cheek fat compartment in a cohort of 50 healthy female subjects. The results analyzed by investigators during the clinical evaluation demonstrated significant amelioration of the FVLS and WSRS. Specifically, a visible amelioration was observed for FVLS in the 3D photographic documentation 4 months after the start of treatment. Investigator clinical evaluations were supported by positive outcomes reported by all patients during the self‐evaluation performed at the end of the study. In particular, subjects self‐reported a marked or very marked improvement in skin firmness and a reduction of superficial and deep wrinkles. The efficacy of the studied medical device was also more pronounced 4 months after the start of the treatment compared with assessments at earlier time points in the study. These results confirmed the improved duration of stability of the medical device observed in in vitro and preclinical analysis,^28^ suggesting an important role for this product in improving skin defects and the potential long‐term effect of this formulation.

Also, these data are especially important because the product is not a commonly used chemically cross‐linked HA filler. Instead, it is made of hybrid cooperative complexes (HCCs) of high and low molecular weight HA thanks to a thermally induced process (NAHYCO® technology).^22^ Although the process does not involve a chemical cross‐linking process, the molecule showed surprising characteristics, such as a longer resistance to degradation compared to linear HA. In this case, increasing the amount of HA (45 mg of low molecular weight HA and 45 mg of high molecular weight HA in 2 mL) improved the resistance to degradation in a way that was similar to cross‐linked HA fillers.^28^ Although no instrumental investigations were possible, we can assume that the long‐term improvement of FVLS and WSRS scores 4 months after the treatment assessed during the current study is dependent on the lateral cheek fat restoration effect demonstrated by recently published data by Cassuto et al.^29^ In this paper, the authors specifically examined fat restoration in the lateral cheek compartment thanks to US evaluation of skin thickness and fat hyperechoic areas examinations. Taken together, the data proved the efficacy of the investigated medical device to restore adipose tissue, leading to the volume restoration assessed in this current investigation. Moreover, the lipo‐lifting effect observed during the current examination could be possible due to a tissue regenerative effect of the product on the adipose tissue compartment rather than a simple mechanical effect due to a cross‐linked filler. In the future, it would also be useful to test both Profhilo® and Profhilo Structura® in a combined protocol for the same patients, considering the difference in the regenerative activity of the two products. With the bioremodeling effect of Profhilo® in the dermis layer of the skin and the fat restoration effect of Profhilo Structura® in the adipose tissue compartment, a global regenerative lifting effect can be achieved. However, despite the promising efficacy results, the present study also had some limitations due to the lack of instrumental evaluations. In this regard, future studies are required to better evaluate adipose tissue restoration through instrumental analysis, such as ultrasound scans, and to perform evaluations at longer time points. Furthermore, considering the prominent loss of deep fat compartments in the facial malar region during aging, other injection techniques for these areas of the face can be evaluated.^13^

Importantly, the injection of the studied medical device did not cause any serious adverse effects and only light bruises in a few subjects, which were not directly related to the product. The tolerability data confirmed the excellent safety profile of HCC already demonstrated by post‐marketing surveillance analysis^27^, ^30^ performed for Profhilo®.

In conclusion, findings from this study demonstrated the efficacy and tolerability of Profhilo Structura® as a bioremodelling treatment, confirming the potential role of this product to counteract aging‐related alterations within the deep fat compartment of the face. Although further studies are needed to evaluate adipose tissue restoration, this medical device represents a promising treatment to reduce facial volume loss and delay age‐associated skin alterations.

## AUTHOR CONTRIBUTIONS

**Adele Sparavigna**: Conceptualization; data curation; formal analysis; investigation; methodology; validation; writing—review and editing. **Franco Grimolizzi**: Writing—original draft; writing—review and editing. **Clara Cigni**: Writing—original draft; writing—review and editing. **Roberto Lualdi**: Data curation; formal analysis; methodology; writing—review and editing. **Gilberto Bellia**: Writing—original draft; writing—review and editing.

## CONFLICT OF INTEREST STATEMENT

A.S. and R.L. declare no conflict of interest. F.G., C.C., and G.B. are currently IBSA Farmaceutici Italia Srl employees.

## ETHICS STATEMENT

Approval for the study (IBSA Farmaceutici Italia Srl) was previously obtained from a local ethics committee (Protocol number E0421). The single‐center study was conducted at DERMING S.r.l., Clinical Research and Bioengineering Institute, Via Valassina 29, Milano (MI) and performed according to the Declaration of Helsinki. All patients provided informed consent to participate in this study and use their data and photos for scientific research.

## TRANSPARENCY STATEMENT

The lead author Adele Sparavigna affirms that this manuscript is an honest, accurate, and transparent account of the study being reported; that no important aspects of the study have been omitted; and that any discrepancies from the study as planned (and, if relevant, registered) have been explained.

## Data Availability

The data presented in this study are available on request from the corresponding author.
